# Circulating microRNA-125b Levels Are Associated With the Risk of Vascular Calcification in Healthy Community-Dwelling Older Adults

**DOI:** 10.3389/fcvm.2021.624313

**Published:** 2021-02-22

**Authors:** Chia-Ter Chao, Der-Sheng Han, Jenq-Wen Huang

**Affiliations:** ^1^Nephrology Division, Department of Internal Medicine, National Taiwan University Hospital BeiHu Branch, Taipei, Taiwan; ^2^Graduate Institute of Toxicology, National Taiwan University College of Medicine, Taipei, Taiwan; ^3^Geriatric and Community Medicine Research Center, National Taiwan University Hospital BeiHu Branch, Taipei, Taiwan; ^4^Department of Rehabilitation and Physical Medicine, National Taiwan University Hospital BeiHu Branch, Taipei, Taiwan; ^5^Nephrology Division, Department of Internal Medicine, National Taiwan University Hospital Yunlin Branch, Yunlin, Taiwan

**Keywords:** aortic calcification, biomarker, chronic kidney disease, epigenetics, geriatrics, microRNA, miR-125b, vascular calcification

## Abstract

**Background:** Vascular calcification (VC) is a subclinical manifestation of vascular disease burden among older adults, conferring an elevated mortality risk. Biomarkers capable of detecting and risk-stratifying VC associated with advanced age remains unavailable, impeding our effort to provide optimal care to geriatric patients.

**Objectives:** In this study, we aimed to investigate whether circulating miR-125b served as a potential indicator for VC in relatively healthy older adults.

**Methods:** Community-dwelling older adults (age ≥65) were prospectively recruited during 2017, followed by clinical features documentation and VC rating based on aortic arch calcification (AAC) and abdominal aortic calcification (AbAC). Multiple logistic regression was done to evaluate the relationship between circulating miR-125b levels, VC presence and severity, followed by selecting the optimal cutoff point for VC diagnosis.

**Results:** A total of 343 relatively healthy older adults (median age, 73.8 years; 40% male; 59.8% having AAC) were enrolled, with a median circulating miR-125b level of 0.012 (interquartile range, 0.003–0.037). Those with more severe AAC had progressively decreasing miR-125b levels (*p*<0.001). Multiple regression analyses showed that having higher miR-125b levels based on the median value were associated with a substantially lower risk of AAC [odds ratio (OR) 0.022, 95% confidence interval (CI) 0.011–0.044] compared to those having lower ones. An optimal cutoff of miR-125b for identifying AAC in older adults was 0.008, with a sensitivity and specificity of 0.86 and 0.80, respectively. Similar findings were obtained when using AbAC as the endpoint.

**Conclusions:** We found that miR-125b serves as an independent indicator for VC in relatively healthy older adults, and may potentially be linked with VC pathophysiology.

## Introduction

Traditional cardiovascular risk factors include several morbidities [hypertension, diabetes mellitus (DM), and hypercholesterolemia] and a higher age. An increased age *per se* contributes to unfavorable morphological and functional changes involving cardiovascular tissues including the myocardium and the major vessels (1), culminating in a significantly elevated risk of major adverse cardiac events (MACEs) compared to their younger counterparts (2). Age also plays an integral role in the estimation of 10-year atherosclerotic cardiovascular disease (ASCVD) risk as endorsed by major professional societies (3). Importantly, chronological aging places affected individuals at an increased risk of vascular calcification (VC), which involves the ectopic deposition of calcium apatite in the vascular wall resembling osteogenesis. Furthermore, the presence of VC has been found to accelerate the progress of vascular inflammation as patients get older (4), and the combination of VC and vascular aging/inflammation increases the future risk of cardiovascular mortality. From this perspective, an in-depth understanding of the pathogenesis of aging-related VC and more instrumentally, how to identify those at risk of developing this vascular morbidity assumes importance in this era of population aging.

The pathophysiology of VC comprises of passive calcium deposition surrounding a mineralization core and the active osteoid-like substance secretion from phenotypically switched resident cells within vascular wall. Epigenetic processes, especially non-coding RNAs, have been implicated as vital players during the course of VC, shaping its initiation and propagation process (5). Among the purview of non-coding RNAs, microRNAs (miRNAs) are important members that influence the risk of VC through modulating the osteoblastic differentiation tendency of vascular smooth muscle cells (VSMCs) (5). Specifically, miR-125b has been the prototype with comprehensive evidence supporting its antagonistic effect against VC; Goettsch and colleagues first disclosed that miR-125b repressed VSMC calcification *in vitro* nearly a decade ago (6). Subsequent studies further unveil the versatile role of miR-125b in vascular pathologies involving endothelial cells, VSMCs, and infiltrating macrophages (7). Judging from the pathophysiological importance of miR-125b in VC and the feasibility of detecting miRNAs in biological fluids, it is tempting to speculate whether miR-125b can assist in diagnosing and risk stratifying VC in the clinical setting. We previously showed that among 223 patients without and with uremic VC, circulating miR-125b inversely correlated with VC severity and its baseline levels could effectively predict the risk of VC worsening in the future (8). Moreover, VC related to chronic kidney disease (CKD) and to aging may share similarities in risk factors and pathologies (9). Consequently, our previous results inspire us to hypothesize that circulating miR-125b levels may exhibit similar associations with the severity of aging-related VC in older adults. We harnessed a prospectively enrolled cohort of community-dwelling older adults to examine this hypothesis.

## Methods

### Ethical Statement

The study protocol has been approved by the institutional review board of National Taiwan University Hospital (No. 201601091RIND). The protocol adhered to the Declaration of Helsinki, and all participants provided written informed consent.

### Recruitment of Study Participants

The study protocol has been published previously (10). In brief, community-dwelling older adults (age ≥65) were prospectively recruited from health examination programs and clinics of National Taiwan University Hospital BeiHu Branch as well as Taipei municipal long-term care service centers during 2017. Exclusion criteria consisted of those who could not communicate in a conscious state. After enrollment, participants were instructed to complete a dedicated questionnaire documenting their sociodemographic profile, self-report comorbidities, and current medication regimens. Following the recording of clinical features, we measured their anthropometric parameters and physical indices [blood pressure (BP) and heart rate (HR)]. Under the fasting status, participants received 10 mL of blood drawing and dipstick urinalysis. We assayed their complete hemogram and serum biochemistry (nutrition, lipid profile, glucose, renal function, and inflammation-related parameters). Participants' estimated glomerular filtration rate (eGFR) was calculated using the four-variable Modification of Diet in Renal Disease (MDRD) formula.

### Vascular Calcification Semi-quantification

We adopted two approaches for semi-quantitatively examining the presence and the extent of VC, aortic arch calcification (AAC) and abdominal aorta calcification (AbAC), based on our prior work (8, 11, 12) and the existing literature (13, 14). A majority of the participants received posteroanterior chest radiography after documenting their baseline clinical features. These films were identified with results rated semi-quantitatively as having no AAC, and having category 1, 2, and 3 AAC. Some of the participants received lateral lumbar spine radiography, with AbAC rated based on the well-validated Kauppila scores (range 0–24, higher scores meaning more severe calcification) (15, 16). Image interpretation was performed by two researchers (CTC and JWH) with an excellent consistency, while controversies were resolved by another physician.

### Quantification of Circulating miR-125b Levels

Part of the collected blood was centrifuged with plasma cryopreserved. Existing reports suggest that circulating miRNAs remain stable in stored sera/plasma without repetitive freeze-thaw cycles (17). First-time thawed sera were subject to cell-free small RNA extraction using the miRNeasy Serum/Plasma kit (QIAGEN, Netherland), which has been credited for enriching low abundant levels of miRNAs especially in biological fluids (18), according to the manufacturer's instruction. The procedure of reverse transcription (RT) and quantitative polymerase chain reaction (qPCR) was briefly described below. A maximal of 2 μg extracted RNA was mixed with miScript Reverse Transcriptase Mix, HiSpec Buffer, and miScript Nucleics Mix (all from miScript PCR System; QIAGEN, Netherland) in a fixed ratio. Mixtures were centrifuged and subsequently incubated at 37°C for 60 min, at 95°C for 5 min to obtain cDNAs. A maximal of 3 ng cDNA per reaction were then diluted in RNase-free water, mixed with miScript Universal Primer, QuantiTect SYBR Green PCR master mix, and the primers of miR-125b (Cat No. MS00006629; QIAGEN, Netherland) or those of *Caenorhabditis elegans* miR-39. This was followed by a gentle mix of the mixture, which was dispensed into plates and subjected to real-time PCR cycler (7200 HT thermal cycler, Applied Biosystems, Foster City, CA).

Since studies have shown that U6 is not an appropriate control for quantifying circulating miRNAs (19), we used the spiked-in control, *Caenorhabditis elegans* miR-39, for normalization purpose (18). Results based on this approach reportedly reduce fluctuations in data and permit cross-study comparisons. Data were then averaged with miR-125b levels calculated using the ΔΔCt method (10). If the participants' miR-125b expression levels were undetectable upon PCR cycling despite the presence of measurable *Caenorhabditis elegans* miR-39, we substituted their miR-125b cycle number with 40, which was the lowest detectable value specified by the cycler, followed by calculation.

### Statistical Analysis

We first used the Kolmogorov-Smirnov test to determine whether the collected continuous variables and circulating miR-125b levels were parametric. For parametric and non-parametric ones, we used means ± standard deviations and medians with interquartile ranges for expression, respectively, while for categorical variables, we used numbers with percentages in parentheses for expression. Comparisons between 2 groups of parametric or non-parametric variables were done by the Student's *t*-test or Mann-Whitney *U*-test, respectively, while comparisons between categorical ones were made by the chi-square test. We used one-way analysis of variance and Kruskal-Wallis test to compare parametric and non-parametric variables of >2 groups, respectively.

We first compared participants' clinical features and laboratory profiles between those with and without AAC and between those with different AAC severities. We also examined whether circulating miR-125b levels differed depending on VC. This was followed by multiple regression analyses with stepwise backward variable selection, with AAC presence as the dependent variable, incorporating variables with a *p* < 0.1 in univariate analysis, and miR-125b. MiR-125b levels were accounted for in the regression models in different styles, as a continuous variable or binarily divided based on the mean or median value. We further used the receiver-operating characteristics (ROC) curves to evaluate the performance of each regression model, followed by the calculation of the area under ROC curves (AUROCs). Youden's index was utilized to capture the optimal cutoff of circulating miR-125b to identify AAC, followed by the comparison of clinical features between those with ≥ and < the cutoff value and a repeated regression analysis.

Several sensitivity analyses were planned beforehand. First, we incorporated miR-125b levels in tertiles or quartiles into the multiple regression models with VC status as the dependent variables, incorporating the same set of variables as described above, with AUROC obtained. In addition, another set of multiple logistic regression analyses with AbAC as the dependent variable was performed, incorporating variables with a *p* < 0.1 between those with and without AbAC.

## Results

Totally 384 community-dwelling older people were recruited during the study period, among whom 41 (10.7%) did not receive chest radiography, leaving 343 (89.3%) in the analysis. No significant difference in demographic distribution was noted between included and excluded ones. The median age of these 343 older adults were 73.8 (68.6–78.6) years, with 40% male. Most participants were morbidity-free, with less than half having hypertension (43%), coronary artery disease (18%), and DM (11%) (Table 1). Among them, 205 (59.8%) had AAC. Those with AAC had a significantly higher age (*p* = 0.008), more likely to have DM (*p* = 0.004) and received anti-diabetic medications (*p* = 0.003), and had significantly lower hemoglobin (*p* = 0.022) but higher globulin (*p* = 0.032) and glucose (*p* = 0.01) than those without (Table 1). Specifically, a greater severity of AAC was paralleled by an increasing age (*p* < 0.001), a higher prevalence of DM (*p* = 0.002) and using anti-diabetics (*p* = 0.001), and a higher fasting glucose (*p* = 0.033) (Table 1).

**Table 1:** Comparison of features between community-dwelling older adults with and without different AAC severities.

	**Total** ** (*n* = 343)**	**Without AAC** ** (*n* = 138)**	**With AAC** ** (*n* = 205)**	***p-value***	**AAC Cat. 1** ** (*n* = 133)**	**AAC Cat. 2** ** (*n* = 54)**	**AAC Cat. 3** ** (*n* = 18)**	***p-value***
Sociodemographic factors^*^								
Age (years)	73.8 (68.6, 78.6)	71.0 (67.7, 77.7)	74.7 (69.5, 78.8)	0.008	74.2 (68.7, 77.6)	74.6 (69.6, 79.5)	79.6 (75.0, 81.8)	<0.001
Sex (male %)	138 (40)	61 (44)	77 (38)	0.220	53 (40)	19 (35)	5 (28)	0.452
Regular exercise (%)	123 (36)	50 (36)	73 (36)	0.125	105 (79)	47 (87)	15 (83)	0.234
Regular alcohol (%)	75 (22)	35 (25)	40 (20)	0.200	22 (17)	15 (28)	3 (17)	0.206
Smoking (%)	11 (3)	2 (1)	9 (4)	0.130	7 (5)	1 (2)	1 (6)	0.283
**Comorbidities**								
DM (%)	38 (11)	7 (5)	31 (15)	0.004	18 (14)	7 (13)	6 (33)	0.002
HTN (%)	147 (43)	53 (38)	94 (46)	0.173	57 (43)	26 (48)	11 (61)	0.244
Hyperlipidemia (%)	67 (20)	21 (15)	46 (22)	0.099	30 (23)	11 (20)	5 (28)	0.363
CAD (%)	63 (18)	25 (18)	38 (19)	0.922	26 (20)	7 (13)	5 (28)	0.526
Prior CVA (%)	10 (3)	3 (2)	7 (3)	0.504	6 (5)	1 (2)	0 (0)	0.531
Gout (%)	16 (5)	6 (4)	10 (5)	0.820	4 (3)	3 (6)	3 (17)	0.079
PUD (%)	62 (18)	25 (18)	37 (18)	0.987	25 (19)	6 (11)	6 (33)	0.201
CLD (%)	29 (8)	14 (10)	15 (7)	0.357	10 (8)	4 (7)	1 (6)	0.820
CKD (%)	12 (4)	5 (4)	7 (3)	0.918	4 (3)	1 (2)	2 (11)	0.307
Prostatic hyperplasia (%)	67 (20)	24 (17)	43 (21)	0.413	30 (23)	9 (17)	4 (22)	0.676
Chronic lung disease (%)	17 (5)	8 (6)	9 (4)	0.557	7 (5)	1 (2)	1 (6)	0.719
Malignancy (%)	18 (5)	9 (7)	9 (4)	0.387	7 (5)	1 (2)	1 (6)	0.638
**Regular medications**								
Anti-HTN (%)	140 (41)	52 (38)	88 (43)	0.334	53 (40)	25 (46)	10 (56)	0.410
Anti-PLT/anti-coagulant (%)	62 (18)	24 (17)	38 (19)	0.788	27 (20)	7 (13)	4 (22)	0.651
Anti-DM (%)	35 (10)	6 (4)	29 (14)	0.003	16 (13)	7 (13)	6 (33)	0.001
Anti-lipid (%)	52 (15)	17 (12)	35 (17)	0.230	23 (17)	9 (17)	3 (17)	0.693
Physical parameters^*^								
SBP (mmHg)	128.8 ± 16.2	127.9 ± 15.0	129.4 ± 16.9	0.376	128.0 ± 15.9	131.8 ± 17.5	133.1 ± 22.1	0.276
DBP (mmHg)	74.0 ± 9.6	75.2 ± 9.1	73.2 ± 9.9	0.056	73.0 ± 9.2	73.8 ± 9.3	73.3 ± 16.0	0.271
HR (min)	69 (63, 76)	68 (64, 77)	70 (63, 76)	0.739	70 (63, 75)	72 (62, 77)	67 (62, 77.5)	0.761
BMI (kg/m^2^)	23.4 (21.4, 25.5)	23.4 (21.4, 25.6)	23.5 (21.5, 25.5)	0.901	23.5 (21.5, 25.7)	23.3 (21.1, 25.2)	24.4 (20.7, 28.1)	0.817
WC (cm)	81.4 ± 9.6	81.6 ± 9.6	81.2 ± 9.6	0.699	81.5 ± 10.3	80.0 ± 8.0	82.2 ± 9.8	0.729
**Urinalysis results**								
Hematuria (%)	123 (36)	50 (36)	73 (36)	0.907	52 (39)	16 (30)	5 (28)	0.568
Proteinuria (%)	38 (11)	20 (14)	18 (9)	0.099	13 (10)	2 (4)	3 (17)	0.145
Hemogram^*^								
WBC (K/μL)	5.1 (4.3, 6.2)	4.9 (4.3, 6.0)	5.2 (4.3, 6.3)	0.305	5.4 (4.3, 6.4)	4.9 (4.2, 5.7)	5.9 (4.7, 6.7)	0.077
PLT (K/μL)	209 (174, 246)	214 (179, 244)	202 (172, 248)	0.433	209 (177, 257)	199 (166, 232)	210 (161, 249)	0.736
Hemoglobin (g/dL)	13.6 (12.9, 14.4)	13.8 (13.1, 14.8)	13.5 (12.7, 14.2)	0.022	13.4 (12.7, 14.2)	13.7 (13.0, 14.4)	13.5 (12.6, 14.1)	0.064
MCV (fL)	92.7 (90.0, 95.5)	92.8 (89.9, 95.3)	92.7 (90.3, 95.9)	0.593	93.0 (90.5, 96.1)	92.0 (89.2, 94.8)	92.3 (88.4, 96.4)	0.472
RDW (%)	13.1 (12.6, 13.6)	12.9 (12.6, 13.5)	13.2 (12.6, 13.7)	0.207	13.1 (12.6, 13.6)	13.3 (12.5, 13.7)	13.4 (12.6, 14.3)	0.381
Renal function^*^								
BUN (mg/dL)	16.1 (13.5, 19.5)	16.6 (13.5, 19.5)	15.9 (13.4, 19.4)	0.390	16.1 (13.4, 19.4)	15.0 (13.3, 19.2)	17.1 (13.8, 19.5)	0.633
Creatinine (mg/dL)	0.8 (0.6, 0.9)	0.8 (0.6, 0.9)	0.8 (0.6, 0.9)	0.632	0.8 (0.6, 0.9)	0.7 (0.6, 0.9)	0.8 (0.6, 0.8)	0.663
eGFR (mL/min/1.73 m^2^)	88.9 (75.8, 104.2)	89.1 (76.7, 104.3)	88.7 (75.5, 104.2)	0.549	88.7 (75.5, 103.8)	88.8 (75.8, 105.8)	86.0 (74.5, 103.2)	0.698
Metabolic profile^*^								
Albumin (mg/dL)	4.3 (4.1, 4.4)	4.3 (4.1, 4.4)	4.3 (4.1, 4.4)	0.534	4.2 (4.1, 4.4)	4.3 (4.2, 4.4)	4.3 (4.1, 4.4)	0.933
Globulin (mg/dL)	2.8 (2.6, 3.0)	2.7 (2.5, 2.9)	2.8 (2.6, 3.0)	0.032	2.8 (2.6, 3.0)	2.8 (2.7, 3.0)	2.7 (2.5, 3.1)	0.115
A/G ratio	1.5 (1.4, 1.7)	1.6 (1.4, 1.7)	1.5 (1.4, 1.7)	0.101	1.5 (1.4, 1.7)	1.5 (1.4, 1.7)	1.7 (1.3, 1.8)	0.210
Glucose (mg/dL)	95 (89, 103)	93 (87, 100)	96 (89, 104)	0.010	96 (89, 104)	97 (89, 103)	102 (91, 120)	0.033
Uric acid (mg/dL)	5.5 ± 1.2	5.6 ± 1.2	5.5 ± 1.2	0.280	5.5 ± 1.2	5.4 ± 1.1	5.5 ± 0.9	0.719
TC (mg/dL)	189 (163, 204)	189 (166, 206)	188 (162, 204)	0.574	181 (160, 201)	194 (171, 217)	189 (172, 211)	0.083
TG (mg/dL)	95 (70, 129)	95 (71, 128)	95 (70, 134)	0.827	92 (68, 135)	103 (78, 132)	94 (62, 124)	0.531
LDL cholesterol (mg/dL)	108 (89, 124)	109 (91, 123)	108 (88, 125)	0.618	105 (85, 120)	115 (96, 140)	104 (88, 128)	0.083
HDL cholesterol (mg/dL)	55 (46, 68)	57 (47, 68)	54 (46, 67)	0.473	54 (45, 67)	56 (48, 68)	58 (46, 70)	0.837

* *Continuous data are expressed in medians with interquartile ranges (if non-parametric) or means ± standard deviations (if parametric)*.

*AAC, aortic arch calcification; A/G, albumin to globulin; BMI, body mass index; BUN, blood urea nitrogen; CAD, coronary artery disease; Cat, category; CKD, chronic kidney disease; CLD, chronic liver disease; CVA, cerebrovascular accident; DBP, diastolic blood pressure; DM, diabetes mellitus; eGFR, estimated glomerular filtration rate based on the Modification of Diet in Renal Disease (MDRD) formula; HDL, high density lipoprotein; HR, heart rate; HTN, hypertension; LDL, low density lipoprotein; PLT, platelet; PUD, peptic ulcer disease; RDW, red cell distribution width; SBP, systolic blood pressure; TC, total cholesterol; TG, triglyceride; WBC, white blood cell; WC, waist circumference*.

### Distribution and Categorization of miR-125b

The *p*-value of Kolmogorov-Smirnov test of circulating miR-125b levels was <0.001, indicating its non-parametric feature. The mean and median miR-125b values among 343 participants were 0.075 ± 0.3 and 0.012 (0.003, 0.037), respectively. Those with AAC had significantly lower circulating miR-125b than those without [the former vs. the latter, 0.004 (0.002, 0.01) vs. 0.041 (0.02, 0.125); *p* < 0.001]. Participants with a greater AAC severity also had progressively decreased miR-125b levels [without vs. category 1 vs. 2 vs. 3, 0.041 (0.02, 0.125) vs. 0.006 (0.002, 0.013) vs. 0.004 (0.002, 0.007) vs. 0.001 (0.0006, 0.003); *p* < 0.001]. Participants with higher than median miR-125b levels had significantly lower prevalence of DM (*p* = 0.004), less likely to receive anti-diabetics (*p* = 0.005), and less AAC (high vs. low, 24 vs. 93%; *p* < 0.001) compared to those with lower-than median levels.

### Risk Factors for Having AAC in Community-Dwelling Older Adults

**Figure 1:**
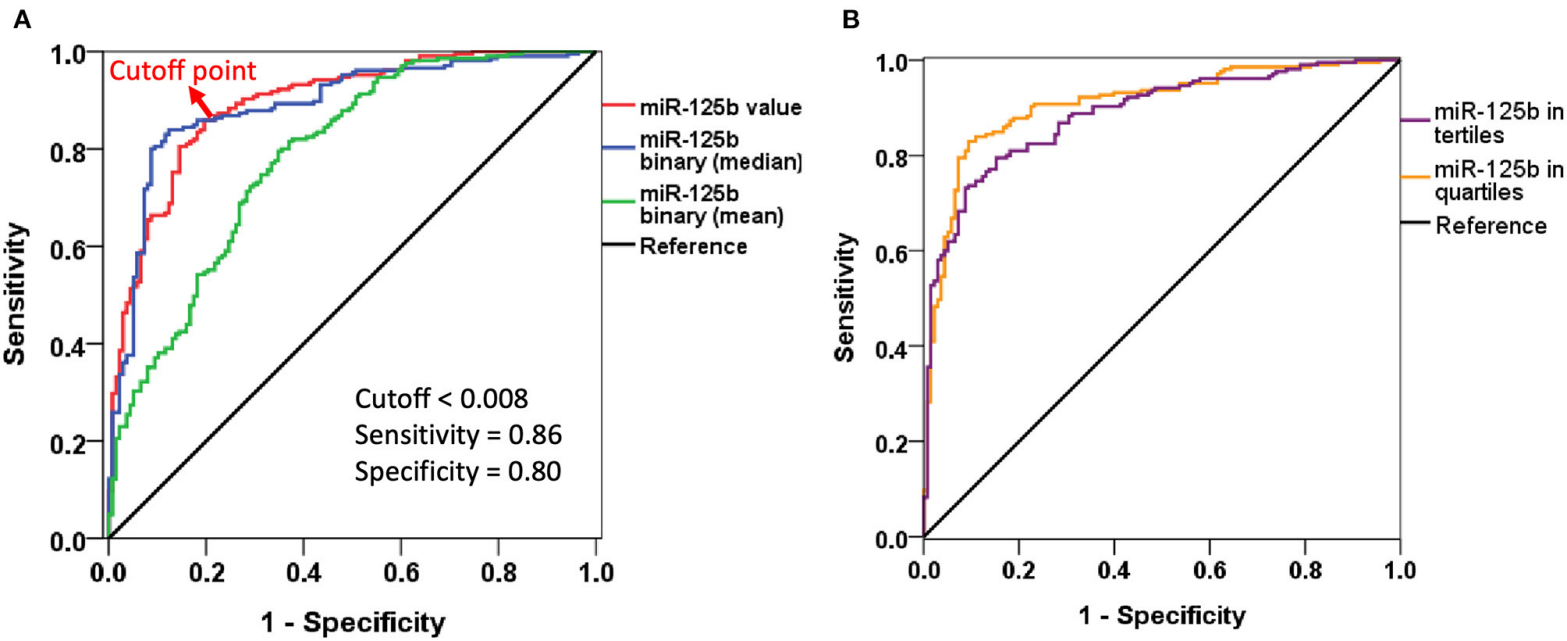
Receiver-operating characteristics curves of different logistic regression models incorporating miR-125b levels. **(A)** Including miR-125b value or in binary division. **(B)** Including miR-125b in tertiles or quartiles.

We subsequently conducted multiple regression analyses to examine risk factors for AAC, incorporating miR-125b in different styles. Regression analyses showed that higher miR-125b levels were significantly associated with a lower probability of having AAC [odds ratio (OR) <0.001 per one unit of miR-125b value, *p* < 0.001], while higher age increased the probability [OR 1.075 per year, 95% confidence interval (CI) 1.023–1.130] (model 1; Table 2). Having higher miR-125b levels based on the mean and median values similarly were associated with a substantially lower risk of having AAC (division based on mean, OR 0.032, 95% CI 0.011–0.094; based on median, OR 0.022, 95% CI 0.011–0.044) (models 2 and 3; Table 2). The AUROCs of models 1, 2, and 3 were 0.893 (95% CI 0.859–0.927), 0.786 (95% CI 0.736–0.835), and 0.890 (95% CI 0.853–0.926), respectively (Figure 1A), supporting the superiority of using the median-based categorization of miR-125b. Youden's index identified that the optimal cutoff of miR-125b level for identifying AAC presence was 0.008, with a sensitivity and specificity of 0.86 and 0.80, respectively (Figure 1A).

**Table 2:** Multiple logistic regression analyses with having AAC as the dependent variable.

	**Odds ratio**	**95% CI**	***p-value***
Model 1: circulating miR-125b as a continuous variable^*^			
Age (per year)	1.075	1.023–1.130	0.004
miR-125b value (per 1 unit)	<0.001^#^	<0.001– <0.001	<0.001
Model 2: circulating miR-125b as a binary variable based on the mean^*^			
Age (per year)	1.052	1.007–1.098	0.023
Use of anti-diabetic medications	3.312	1.177–9.321	0.023
Hemoglobin	0.807	0.657–0.991	0.040
High miR-125b	0.032	0.011–0.094	<0.001
Model 3: circulating miR-125b as a binary variable based on the median^*^			
Age (per year)	1.067	1.015–1.122	0.011
Hyperlipidemia	2.330	1.083–5.013	0.031
High miR-125b	0.022	0.011–0.044	<0.001

*AAC, aortic arch calcification; CI, confidence interval*.

* *Including age, diabetes mellitus, hyperlipidemia, use of anti-diabetic medications, diastolic blood pressure, and laboratory data (proteinuria, globulin, hemoglobin, and fasting glucose), as well as circulating miR-125b*.

# *Very low value relative to the low mean value of miR-125b on average (mean 0.075 ± 0.3)*.

We further divided participants based on the cutoff value 0.008, yielding 195 (56.7%) with high circulating miR-125b levels. Those with a higher-than-cutoff miR-125b had significantly lower prevalence of DM (*p* = 0.003), less likely to receive anti-diabetics (*p* < 0.001), and a lower red cell distribution width (RDW) (*p* = 0.008) than those with lower-than-cutoff values (Supplementary Table 1). Participants with a cutoff-based high miR-125b level were less likely to have AAC and less severe AAC than those without (*p* < 0.001) (Supplementary Table 1). Another multiple logistic regression showed that having a higher-than-cutoff miR-125b was associated with a lower probability of AAC (OR 0.014, 95% CI 0.006–0.038).

### Sensitivity Analysis

After dividing participants based on miR-125b tertiles, we found that participants with an increasing miR-125b tertile also had a progressively lower probability of exhibiting AAC (for tertile 2 and 3 vs. 1, OR 0.062 and 0.007, 95% CI 0.021–0.182 and 0.002–0.021, respectively) than those within the lowest tertile (model S1; Table 3). Similar findings were obtained when we analyzed the risk based on miR-125b quartiles (model S2; Table 3). The AUROCs for models S1 and S2 were 0.887 (95% CI 0.853–0.922) and 0.908 (95% CI 0.876–0.941), respectively (Figure 1B).

**Table 3:** Sensitivity analyses.

	**Odds ratio**	**95% CI**	***p-value***
Model S1: circulating miR-125b in tertiles^*^			
Age (per year)	1.084	1.032–1.138	0.001
Globulin (mg/dL)	2.598	1.009–6.687	0.048
miR-125b tertile 2 vs. 1	0.062	0.021–0.182	<0.001
miR-125b tertile 3 vs. 1	0.007	0.002–0.021	<0.001
Model S2: circulating miR-125b in quartiles^*^			
Age (per year)	1.088	1.031–1.149	0.002
miR-125b quartile 2 vs. 1	0.507	0.140–1.839	0.302
miR-125b quartile 3 vs. 1	0.023	0.007–0.073	<0.001
miR-125b quartile 4 vs. 1	0.007	0.002–0.024	<0.001

*CI, confidence interval*.

* *Including age, diabetes mellitus, hyperlipidemia, use of anti-diabetic medications, diastolic blood pressure, and laboratory data (proteinuria, globulin, hemoglobin, and fasting glucose), as well as circulating miR-125b categories*.

We also examined whether circulating miR-125b levels were associated with AbAC presence and severity. Fifty (14.6%) of participants had lumbar spine films available for evaluation, among whom 19 (38%) and 31 (62%) did not have and had AbAC, respectively. Those with AbAC had significantly lower miR-125b levels than those without [with vs. without, 0.007 (0.002, 0.025) vs. 0.023 (0.01, 0.044); *p* = 0.016]. A multiple logistic regression with stepwise backward variable selection, using AbAC as the dependent variable and incorporating age, sex, DM, globulin, and cutoff-based high miR-125b, showed that having a high circulating miR-125b was similarly associated with a lower risk of AbAC than those without (OR 0.213, 95% CI 0.053–0.852).

## Discussion

In the current study, we prospectively enrolled a moderate sized group of older adults with few morbidities, and tested whether their circulating miR-125b levels exhibited associations with their aortic calcification status and severities. Regression analyses showed that having a high circulating miR-125b level were associated with a lower probability of carrying AAC and AbAC, independent of demographic profiles, morbidities, physical features, and multiple laboratory parameters. Importantly, we identified the cutoff point of miR-125b levels for differentiating between older adults with and without VC, which can be used in subsequent researches. Our findings thus support the notion that miRNAs participate in aging-related VC as well and can serve as a biomarker for diagnosis and even risk stratification.

Prior studies focusing on VC mostly involved patients with DM or CKD, both of which are significant risk enhancers for VC in experimental and clinical reports (20); few specifically address VC predominantly related to aging, while available ones mostly examine coronary artery calcification (CAC). Yano et al. reported that CAC severity predicted incident coronary heart disease and stroke among older adults (21), while Everson-Rose and colleagues identified CAC as a risk factor for impaired walking speed (22). On the other hand, risk factors for aging-related VC in older adults remain elusive and are rarely recognized. An increased waist circumference and age-related loss of lean body mass are independently associated with a higher risk of having renal artery calcification and AbAC, respectively, among community-dwelling older adults (23, 24), while a low bone mineral density and hyperphosphatemia increase the risk of AbAC in the elderly as well (25). However, nearly all available findings harness clinical features or body composition data for risk factor analysis regarding aging-related VC; pathophysiology-based risk factors are rarely examined. In this sense, results from this study substantially extend the existing knowledge by elucidating a novel miRNA-based risk factor for this vascular morbidity; furthermore, we also affirm the clinical utility of this miRNA as a circulating biomarker for estimating the probability of aging-related VC.

The cutoff value of circulating miR-125b we uncovered for recognizing aging-related VC (0.008) differs from that reported previously for uremic VC (0.06–0.07) (8). Prior reports suggested that circulating miRNAs levels decreased as eGFR declined (26), and factors that contribute to the development and worsening of VC frequently intertwine with each other and complicate the pathogenesis, as the degree of renal dysfunction progresses. From this perspective and based on the fact that miR-125b is a negative predictor of VC, the threshold value of miR-125b required for differentiating between the absence and presence of uremic VC may have to be larger than it should be among the general population, in order to better capture those without such illness. Nonetheless, more evidence is needed to clarify the pathophysiological nature of this change in cutoff values.

**Figure 2:**
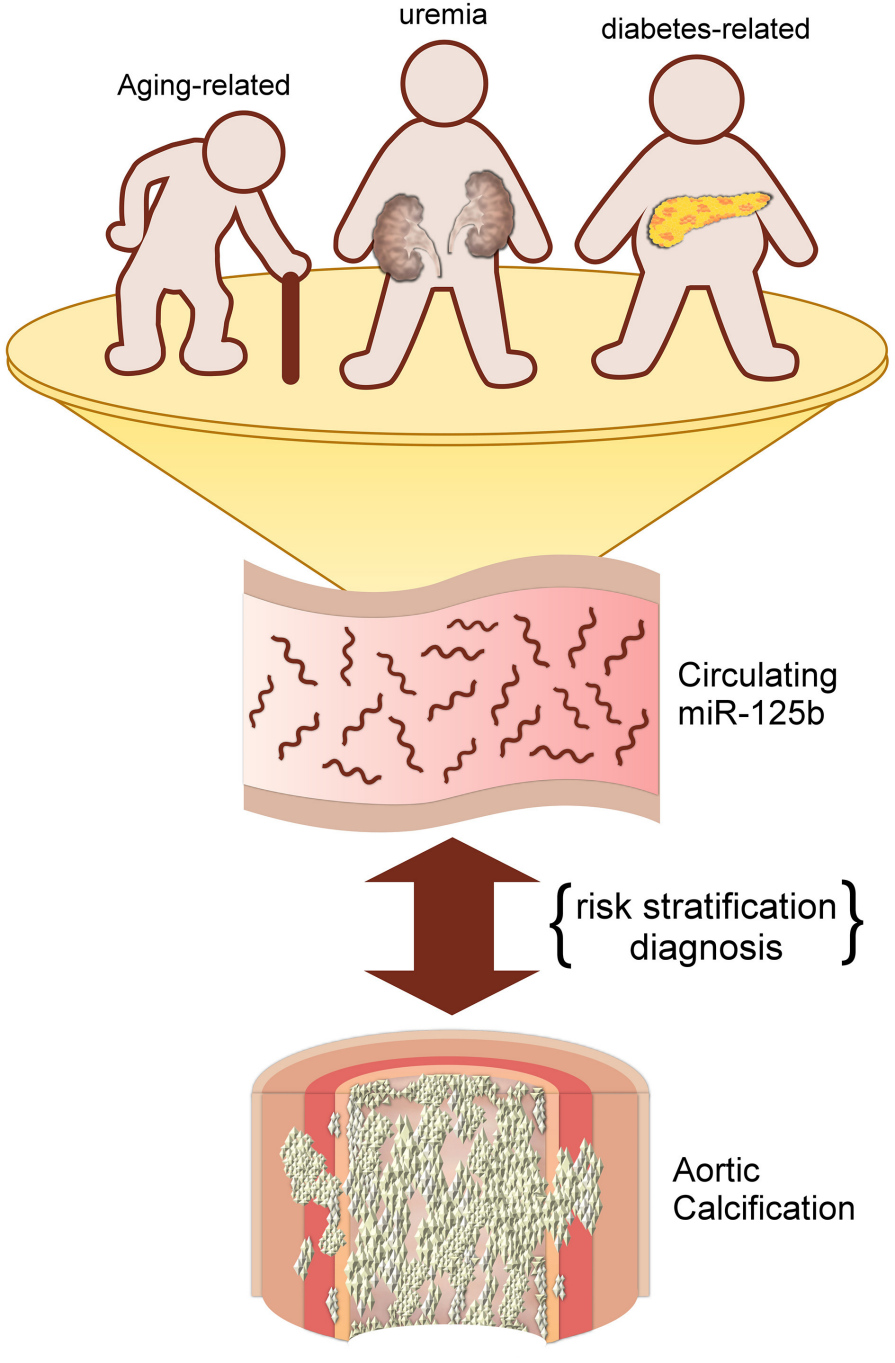
An illustrative diagram depicting the potential utility of circulating miR-125b for predicting aortic calcification/vascular calcification of different origins.

Mechanisms responsible for altering circulating miR-125b levels in those with aging-related VC remain unclear. It has been summarized previously that miR-125b played a pivotal role in attenuating the probability of VSMC trans-differentiation into osteoblast-like cells, thereby decreasing VC tendency (7). Results from *in vitro* experiments affirm that miR-125b participates early during the course of osteogenesis by directly targeting *Cbf*β and indirectly suppressing the effect of *RUNX2*, a vital osteoblast differentiation marker (27). It is thus likely that miR-125b serves as a negative indicator of VC regardless of VC origin. However, we believe that miR-125b participates more deeply in the process of aging-related VC. Biological aging of tissue stem cells has recently been found to down-regulate several competence-regulating miRNAs, one of which is miR-125b (28). The decreased expression of miR-125b following cellular senescence potentially alters tissue responses to environmental signals, leading to abnormal phenotype generation, such as VC. In addition, microRNA levels are known to be affected by medications such as antiplatelet medications (29), and they may also be surrogates of platelet reactivities (30) that potentially influence future cardiovascular risk (31). MiR-125b has been implicated in the pathogenesis of aortic valve calcification (32), a potential surrogate co-existing with AAC (33). Finally, aging is frequently accompanied by the emergence of subclinical chronic inflammation, or “inflammaging,” due to cellular senescence with rising oxidative stress from worn-off mitochondria, inflammasome activation, and immune-dysregulation (34). Lower miR-125b expressions correlate with an age-associated increase in CCL4 levels (35), both of which are involved in the pathogenesis of cardiovascular calcification (32). Based on these findings, the strong association between circulating miR-125b levels and the status and severity of aging-related VC appears reasonable; this relationship has also been affirmed previously for uremic VC and potentially applicable to diabetic VC as well. A pictorial summary of our findings and results from prior literature is provided in Figure 2.

Our study has its strength and limitations. Risk factors and diagnostic biomarkers for aging-related VC are rarely reported in the literature, and our findings greatly extend the utility of circulating miRNAs in triaging VC with regard to the associated risk. Our sample size is adequate, with robust results obtained. However, several issues should be bore in mind before interpreting these data. First, a comprehensive evaluation of aortic calcification severity was not undertaken, and we used semi-quantitative rating schemes to gauge AAC and AbAC extent only. However, the approach we adopted has been repeatedly tested in the existing literature, with results validated in different populations (13–16, 36, 37). Therefore, we believe that our results remain valid. Second, this study did not address the temporal relationship between miR-125b levels and VC progression, so we could not be certain whether miR-125b could foretell the course of aging-related VC in the future. Third, we examined only one microRNA as a marker for VC in this study, whose sensitivity could be limited compared to microRNA combinatorial panels. However, this marker has been validated previously in other population. We are currently in the process of identifying other circulating microRNA candidates for detecting VC. In addition, we did not examine the incidence of aortic valve calcification in our cohort. Finally, there may be other interfering factors that we did not collect or adjust for, such as bone mineral density, body adiposity, and biochemical parameter (serum calcium or phosphate). Since these older adults are relatively health with few morbidities, we believe that these factors are unlikely to influence our findings.

## Conclusion

In conclusion, we prospectively enrolled a group of healthy community-dwelling older adults for analyzing the association between circulating miR-125b and aging-related VC. The close relationship between this miRNA biomarker and the risk/severity of VC serves to inform us that miRNA-based diagnostics may be useful as a non-invasive and radiation-free approach for identifying VC, an important age-related morbidity that increases the risk of adverse outcomes among this ever-rising population.

## Data Availability Statement

The raw data supporting the conclusions of this article will be made available by the authors, subjected to administrative regulations.

## Ethics Statement

The studies involving human participants were reviewed and approved by the institutional review board of the National Taiwan University Hospital (No. 201601091RIND). The patients/participants provided their written informed consent to participate in this study.

## Author Contributions

C-TC and D-SH: study design. C-TC and J-WH: data analysis. C-TC, D-SH, and J-WH: article drafting. All authors approved the final version of the manuscript.

## Conflict of Interest

The authors declare that the research was conducted in the absence of any commercial or financial relationships that could be construed as a potential conflict of interest.

## Abbreviations

AAC: aortic arch calcification
AbAC: abdominal aortic calcification
ASCVD: atherosclerotic cardiovascular disease
AUROC: area under receiver-operating characteristics curve
BP: blood pressure
CI: confidence interval
CKD: chronic kidney disease
DM: diabetes mellitus
eGFR: estimated glomerular filtration rate
HR: heart rate
MACE: major adverse cardiac events
MDRD: Modification of Diet in Renal Disease
miRNA: microRNA
OR: odds ratio
ROC: receiver-operating characteristics
VC: vascular calcification
VSMC: vascular smooth muscle cell.

## References

[1] Letnes JM Nes B Vaardal-Lunde K Slette Martine B Mølmen-Hansen Harald E Aspenes Stian T. Left atrial volume, cardiorespiratory fitness, and diastolic function in healthy individuals: the HUNT study, Norway. J Am Heart Assoc. (2020) 9:e014682. 10.1161/JAHA.119.014682 31986991 PMC7033857

[2] Helber I Alves CMR Grespan SM Veiga ECA Moraes PIM Souza JM. The impact of advanced age on major cardiovascular events and mortality in patients with ST-elevation myocardial infarction undergoing a pharmaco-invasive strategy. Clin Interv Aging. (2020) 15:715–22. 10.2147/CIA.S218827 32546989 PMC7247595

[3] Goff DC Lloyd-Jones Donald M Bennett G Coady S D'Agostino Ralph B Gibbons R. 2013 ACC/AHA guideline on the assessment of cardiovascular risk. Circulation. (2014) 129:S49–73. 10.1161/01.cir.0000437741.48606.98 24222018

[4] Joshi FR Rajani NK Abt M Woodward M Bucerius J Mani V. Does vascular calcification accelerate inflammation?: a substudy of the dal-PLAQUE trial. J Am Coll Cardiol. (2016) 67:69–78. 10.1016/j.jacc.2015.10.050 26764069

[5] Hou Y-C Lu C-L Yuan T-H Liao M-T Chao C-T Lu K-C. The epigenetic landscape of vascular calcification: an integrative perspective. Int J Mol Sci. (2020) 21:980. 10.3390/ijms21030980 32024140 PMC7037112

[6] Goettsch C Rauner M Pacyna N Hempel U Bornstein SR Hofbauer LC. miR-125b regulates calcification of vascular smooth muscle cells. Am J Pathol. (2011) 179:1594–600. 10.1016/j.ajpath.2011.06.016 21806957 PMC3181383

[7] Chao C-T Yeh H-Y Yuan T-H Chiang C-K Chen H-W. MicroRNA-125b in vascular diseases: an updated systematic review of pathogenetic implications and clinical applications. J Cell Mol Med. (2019) 23:5884–94. 10.1111/jcmm.14535 31301111 PMC6714222

[8] Chao C-T Liu Y-P Su S-F Yeh H-Y Chen H-Y Lee P-J. Circulating MicroRNA-125b predicts the presence and progression of uremic vascular calcification. Arteriosc Thromb Vasc Biol. (2017) 37:1402–14. 10.1161/ATVBAHA.117.309566 28522697

[9] Kooman JP Dekker MJ Usvyat LA Kotanko P van der Sande FM Schalkwijk CG. Inflammation and premature aging in advanced chronic kidney disease. Am J Physiol Renal Physiol. (2017) 313:F938–50. 10.1152/ajprenal.00256.2017 28701312

[10] Chao C-T Yeh H-Y Han D-S Huang J-W Huang K-C. Determinants of circulating microRNA-125b, a risk predictor of vascular calcification, among community-dwelling older adults. Clin Transl Med. (2020) 10:e145. 10.1002/ctm2.145 32898327 PMC7423181

[11] Chao C-T Yuan T-H Yeh H-Y Chen H-Y Huang J-W Chen H-W. Risk factors associated with altered circulating micro RNA−125b and their influences on uremic vascular calcification among patients with end-stage renal disease. J Am Heart Assoc. (2019) 8:e010805. 10.1161/JAHA.118.010805 30646802 PMC6497364

[12] Chen S-I Chiang C-L Chao C-T Chiang C-K Huang J-W. Gustatory function and the uremic toxin, phosphate, are modulators of the risk of vascular calcification among patients with chronic kidney disease: a pilot study. Toxins. (2020) 12:420. 10.3390/toxins12060420 32630499 PMC7354456

[13] Hashimoto H Iijima K Hashimoto M Son B-K Ota H Ogawa S. Validity and usefulness of aortic arch calcification in chest X-ray. J Atherosc Thromb. (2009) 16:256–64. 10.5551/jat.E570 19556724

[14] Bannas P Jung C Blanke P Treszl A Derlin T Adam G. Severe aortic arch calcification depicted on chest radiography strongly suggests coronary artery calcification. Eur Radiol. (2013) 23:2652–7. 10.1007/s00330-013-2877-z 23660774

[15] Kiel DP Kauppila LI Cupples LA Hannan MT O'Donnell CJ Wilson PWF. Bone loss and the progression of abdominal aortic calcification over a 25 year period: the Framingham heart study. Calcif Tissue Int. (2001) 68:271–6. 10.1007/BF02390833 11683533

[16] Honkanen E Kauppila L Wikström B Rensma PL Krzesinski J-M Aasarod K. Abdominal aortic calcification in dialysis patients: results of the CORD study. Nephrol Dial Transplant. (2008) 23:4009–15. 10.1093/ndt/gfn403 18676346 PMC2639067

[17] Glinge C Clauss S Boddum K Jabbari R Jabbari J Risgaard B. Stability of circulating blood-based microRNAs - pre-analytic methodological considerations. PLoS ONE. (2017) 12:e0167969. 10.1371/journal.pone.0167969 28151938 PMC5289450

[18] Farina NH Wood ME Perrapato SD Francklyn CS Stein GS Stein JL. Standardizing analysis of circulating microRNA: clinical and biological relevance. J Cell Biochem. (2014) 115:805–11. 10.1002/jcb.24745 24357537 PMC3992702

[19] Xiang M Zeng Y Yang R Xu H Chen Z Zhong J. U6 is not a suitable endogenous control for the quantification of circulating microRNAs. Biochem Biophys Res Commun. (2014) 454:210–4. 10.1016/j.bbrc.2014.10.064 25450382

[20] Krishnan P Moreno PR Turnbull IC Purushothaman M Zafar U Tarricone A. Incremental effects of diabetes mellitus and chronic kidney disease in medial arterial calcification: synergistic pathways for peripheral artery disease progression. Vasc Med. (2019) 24:383–94. 10.1177/1358863X19842276 31090495 PMC9812284

[21] Yano Y O'Donnell CJ Kuller L Kavousi M Erbel R Ning H. Association of coronary artery calcium score vs age with cardiovascular risk in older adults: an analysis of pooled population-based studies. JAMA Cardiol. (2017) 2:986–94. 10.1001/jamacardio.2017.2498 28746709 PMC5710171

[22] Everson-Rose SA Mendes de Leon CF Roetker NS Lutsey PL Alonso A. Subclinical cardiovascular disease and changes in self-reported mobility: multi-ethnic study of atherosclerosis. J Gerontol Biol Sci Med Sci. (2018) 73:218–24. 10.1093/gerona/glx103 28582505 PMC5861943

[23] Ricalde A Allison M Rifkin D Shaw R. Anthropometric measures of obesity and renal artery calcification: results from the Multi-Ethnic Study of Atherosclerosis. Atherosclerosis. (2018) 271:142–7. 10.1016/j.atherosclerosis.2018.02.031 29518746 PMC5870886

[24] Rodríguez AJ Scott D Khan B Khan N Hodge A English DR. Low relative lean mass is associated with increased likelihood of abdominal aortic calcification in community-dwelling older Australians. Calcif Tissue Int. (2016) 99:340–9. 10.1007/s00223-016-0157-z 27272030

[25] Figueiredo CP Rajamannan NM Lopes JB Caparbo VF Takayama L Kuroishi ME. Serum phosphate and hip bone mineral density as additional factors for high vascular calcification scores in a community-dwelling: The São Paulo Ageing & Health Study (SPAH). Bone. (2013) 52:354–9. 10.1016/j.bone.2012.10.019 23098828

[26] Neal CS Michael MZ Pimlott LK Yong TY Li JYZ Gleadle JM. Circulating microRNA expression is reduced in chronic kidney disease. Nephrol Dial Transplant. (2011) 26:3794–802. 10.1093/ndt/gfr485 21891774

[27] Huang K Fu J Zhou W Li W Dong S Yu S. MicroRNA-125b regulates osteogenic differentiation of mesenchymal stem cells by targeting Cbfβ *in vitro*. Biochimie. (2014) 102:47–55. 10.1016/j.biochi.2014.02.005 24560795

[28] Watanabe K Ikuno Y Kakeya Y Kito H Matsubara A Kaneda M. Functional similarities of microRNAs across different types of tissue stem cells in aging. Inflamm Regen. (2018) 38:9. 10.1186/s41232-018-0066-9 29991971 PMC5989452

[29] Carino A De Rosa S Sorrentino S Polimeni A Sabatino J Caiazzo G. Modulation of circulating microRNAs levels during the switch from clopidogrel to ticagrelor. Biomed Res Int. (2016) 2016:3968206. 10.1155/2016/3968206 27366745 PMC4913053

[30] Pordzik J Jakubik D Jarosz-Popek J Wicik Z Eyileten C De Rosa S. Significance of circulating microRNAs in diabetes mellitus type 2 and platelet reactivity: bioinformatic analysis and review. Cardiovasc Diabetol. (2019) 18:113. 10.1186/s12933-019-0918-x 31470851 PMC6716825

[31] Pordzik J Pisarz K De Rosa S Dyve Jones A Eyileten C Indolfi C. The potential role of platelet-related microRNAs in the development of cardiovascular events in high-risk populations, including diabetic patients: a review. Front Endocrinol. (2018) 9:74. 10.3389/fendo.2018.00074 29615970 PMC5869202

[32] Ohukainen P Syväranta S Näpänkangas J Rajamäki K Taskinen P Peltonen T. MicroRNA-125b and chemokine CCL4 expression are associated with calcific aortic valve disease. Ann Med. (2015) 47:423–9. 10.3109/07853890.2015.1059955 26203686

[33] Sabatino J Wicik Z De Rosa S Eyileten C Jakubik D Spaccarotella C. MicroRNAs fingerprint of bicuspid aortic vale. J Mol Cell Cardiol. (2019) 134:98–106. 10.1016/j.yjmcc.2019.07.001 31278905

[34] Ferrucci L Fabbri E. Inflammageing: chronic inflammation in ageing, cardiovascular disease, and frailty. Nat Rev Cardiol. (2018) 15:505–22. 10.1038/s41569-018-0064-2 30065258 PMC6146930

[35] Cheng N-L Chen X Kim J Shi AH Nguyen C Wersto R. MicroRNA-125b modulates inflammatory chemokine CCL4 expression in immune cells and its reduction causes CCL4 increase with age. Aging Cell. (2015) 14:200–8. 10.1111/acel.12294 25620312 PMC4364832

[36] Lee ST Chao CT Huang JW Huang LC. Vascular calcification as an underrecognized risk factor for frailty in 1783 community-dwelling elderly individuals. J Am Heart Assoc. (2020) 9:e017308. 10.1161/JAHA.120.017308 32875940 PMC7727009

[37] Chao CT Yeh HY Tsai YT Chiang CK Chen HW. A combined microRNA and target protein-based panel for predicting the probability and severity of uremic vascular calcification: a translational study. Cardiovasc Res. (2020) cvaa255. 10.1093/cvr/cvaa255 32866261

