# Supplementary material for: Circulating microRNA-125b Levels Are Associated With the Risk of Vascular Calcification in Healthy Community-Dwelling Older Adults
Source: Front Cardiovasc Med. 2021 Feb 22;8:624313. doi: 10.3389/fcvm.2021.624313 (PMC7937626; doi:10.3389/fcvm.2021.624313)
Supplement: Supplementary file 1 [file Table_1.doc]

**Supplementary Table 1. Comparison of features between participants with a circulating miR-125b levels higher than or equal to and lower than the cutoff value**

|  | ≥ cutoff **(n = 195)** | **< cutoff**  **(n = 148)** | ***p-value*** |
| --- | --- | --- | --- |
|  |
| *Sociodemographic factors** |  |  |  |
| **Age (years)** | 72.7 (68.4, 78.6) | 74.2 (68.9, 78.7) | *0.548* |
| **Sex (male %)** | 82 (42) | 56 (38) | *0.432* |
| **Regular exercise (%)** | 163 (84) | 125 (84) | *0.828* |
| **Regular alcohol (%)** | 48 (25) | 27 (18) | *0.158* |
| **Smoking (%)** | 4 (2) | 7 (5) | *0.164* |
| *Comorbidities* |  |  |  |
| **DM (%)** | 13 (7) | 25 (17) | *0.003* |
| **HTN (%)** | 85 (44) | 62 (42) | *0.754* |
| **Hyperlipidemia (%)** | 38 (19) | 29 (20) | *0.980* |
| **CAD (%)** | 35 (18) | 28 (19) | *0.819* |
| **Prior CVA (%)** | 5 (3) | 5 (3) | *0.658* |
| **Gout (%)** | 10 (5) | 6 (4) | *0.642* |
| **PUD (%)** | 37 (19) | 25 (17) | *0.621* |
| **CLD (%)** | 15 (8) | 14 (9) | *0.561* |
| **CKD (%)** | 6 (3) | 6 (4) | *0.627* |
| **Prostatic hyperplasia (%)** | 34 (17) | 33 (22) | *0.262* |
| **Thyroid disorder (%)** | 30 (15) | 18 (12) | *0.396* |
| **Chronic lung disease (%)** | 11 (6) | 6 (4) | *0.504* |
| **Malignancy (%)** | 13 (7) | 5 (3) | *0.177* |
| *Regular medications* |  |  |  |
| **Anti-HTN (%)** | 82 (42) | 58 (39) | *0.595* |
| **Anti-PLT/anti-coagulant (%)** | 35 (18) | 27 (18) | *0.944* |
| **Anti-DM (%)** | 11 (6) | 24 (16) | *<0.001* |
| **Anti-lipid (%)** | 31 (16) | 21 (14) | *0.663* |
| *Physical parameters** |  |  |  |
| **SBP (mmHg)** | 129.8 ± 15.1 | 127.4 ± 17.4 | *0.173* |
| **DBP (mmHg)** | 74.6 ± 9.2 | 73.2 ± 10.2 | *0.197* |
| **HR (/min)** | 69 (63, 76) | 70 (63, 77) | *0.927* |
| **BMI (kg/m^2^)** | 23.5 (21.5, 25.9) | 23.1 (21.4, 25.3) | *0.377* |
| **WC (cm)** | 81.9 ± 9.9 | 80.6 ± 9.2 | *0.203* |
| *Urinalysis results* |  |  |  |
| **Hematuria (%)** | 71 (36) | 52 (35) | *0.808* |
| **Proteinuria (%)** | 24 (12) | 14 (9) | *0.407* |
| *Hemogram** |  |  |  |
| **WBC (K/μL)** | 5.1 (4.3, 6.1) | 5.1 (4.2, 6.2) | *0.702* |
| **PLT (K/μL)** | 212 (177, 245) | 199 (169, 247) | *0.346* |
| **Hemoglobin (g/dL)** | 13.7 (13.0, 14.6) | 13.5 (12.7, 14.2) | *0.085* |
| **MCV (fL)** | 93.0 (90.6, 95.5) | 92.2 (89.3, 95.5) | *0.115* |
| **RDW (%)** | 13.0 (12.5, 13.4) | 13.3 (12.6, 13.8) | *0.008* |
| *Renal function** |  |  |  |
| **BUN (mg/dL)** | 16.5 (13.7, 19.7) | 15.7 (13.3, 18.6) | *0.104* |
| **Creatinine (mg/dL)** | 0.8 (0.6, 0.9) | 0.8 (0.6, 0.9) | *0.620* |
| **eGFR (mL/min/1.73 m^2^)** | 88.9 (76.3, 104.7) | 88.7 (75.8, 103.6) | *0.775* |
| *Metabolic profile** |  |  |  |
| **Albumin (mg/dL)** | 4.3 (4.1, 4.4) | 4.3 (4.2, 4.4) | *0.370* |
| **Globulin (mg/dL)** | 2.8 (2.6, 2.9) | 2.8 (2.5, 3.0) | *0.285* |
| **A/G ratio** | 1.5 (1.4, 1.7) | 1.6 (1.4, 1.7) | *0.972* |
| **Glucose (mg/dL)** | 95 (88, 102) | 97 (90, 104) | *0.076* |
| **Uric acid (mg/dL)** | 5.5 ± 1.2 | 5.6 ± 1.1 | *0.944* |
| **TC (mg/dL)** | 187 (161, 203) | 190 (167, 206) | *0.442* |
| **TG (mg/dL)** | 94 (71, 128) | 96 (69, 134) | *0.975* |
| **LDL cholesterol (mg/dL)** | 108 (88, 124) | 108 (92, 124) | *0.664* |
| **HDL cholesterol (mg/dL)** | 56 (46, 64) | 55 (46, 69) | *0.660* |
| *AAC status and severity* |  |  |  |
| **No AAC** | 133 (68) | 5 (3) | *<0.001* |
| **AAC presence** | 62 (32) | 143 (97) |  |
| Category 1 | 51 (26) | 82 (55) |  |
| Category 2 | 11 (6) | 43 (29) |  |
| Category 3 | 0 (0) | 18 (12) |  |

**Continuous data are expressed in median with interquartile ranges (if non-parametric) or mean +/- standard deviations (if parametric)*

*AAC, aortic arch calcification; A/G, albumin to globulin; BMI, body mass index; BUN, blood urea nitrogen; CAD, coronary artery disease; Cat, category; CKD, chronic kidney disease; CLD, chronic liver disease; CVA, cerebrovascular accident; DBP, diastolic blood pressure; DM, diabetes mellitus; eGFR, estimated glomerular filtration rate based on the Modification of Diet in Renal Disease (MDRD) formula; HDL, high density lipoprotein; HR, heart rate; HTN, hypertension; LDL, low density lipoprotein; PLT, platelet; PUD, peptic ulcer disease; RDW, red cell distribution width; SBP, systolic blood pressure; TC, total cholesterol; TG, triglyceride; WBC, white blood cell; WC, waist circumference*
